# Editorial: Adenylyl cyclase isoforms as potential drug targets

**DOI:** 10.3389/fphar.2022.1098240

**Published:** 2022-12-09

**Authors:** Tarsis F. Brust, Rennolds S. Ostrom, Val J. Watts

**Affiliations:** ^1^ Department of Pharmaceutical Sciences, Lloyd L. Gregory School of Pharmacy, Palm Beach Atlantic University, West Palm Beach, FL, United States; ^2^ ATyr Pharma, San Diego, CA, United States; ^3^ Department of Biomedical and Pharmaceutical Sciences, Chapman University School of Pharmacy, Irvine, CA, United States; ^4^ Department of Medicinal Chemistry and Molecular Pharmacology, Purdue University, West Lafayette, IN, United States

**Keywords:** adenylyl cyclase, cAMP, adenylate cyclase, G protein, cell signaling

Adenylyl cyclases (ACs) are important signaling enzymes that catalyze the conversion of adenosine triphosphate (ATP) into the second messenger, cyclic adenosine monophosphate (cAMP). cAMP has numerous cellular functions that translate to physiological outcomes. The ACs are diverse with 10 isoforms that are modulated through numerous different mechanisms (Ostrom et al., 2022). For instance, activation of G protein-coupled receptors (GPCRs), which are targeted by nearly one-third of all FDA-approved drugs, modulates the activity ACs directly through G protein subunits as well as through second messenger signaling pathways (Ostrom et al., 2022; Santos et al., 2017). Thus, it is surprising that although numerous drugs indirectly modulate AC activity, there are no drugs in the market that were designed to directly modulate AC isoforms. The goal of this Research Topic is to highlight and compile recent efforts implicating the development of therapeutic strategies that target AC isoforms.

The Research Topic has nine different articles. A common topic on all four original research articles is adenylyl cyclase 1 (AC1). Two of those articles highlight the role of AC1 in pain and nociception. Giacoletti et al. show that the selective AC1 inhibitor ST034307 (Brust et al., 2017) is efficacious in several different mouse pain models. Johnson et al. also use ST034307 and AC1 knockdown to show that both strategies for reducing AC1 activity lead to analgesic effects and a reduction of morphine-induced hyperalgesia in mice. These articles also show that inhibition/knockdown of AC1 does not cause analgesic tolerance or major disruptions of normal mouse behaviors. The article by Dwyer et al. focuses on novel strategies for the discovery of AC1 inhibitors. The authors report new small molecule scaffolds for AC1 inhibitors and also provide SAR information for tuning AC1/AC8 selectivity and inhibitory potency. The fourth original research article, by Bose et al., focuses on the role of AC1 in the sino-atrial node to regulate heart rate. The authors also use ST034307 and show that AC1 inhibition reduces the positive chronotropic effects of phenylephrine in tissue preparations from guinea pigs. These articles highlight the therapeutic potential of small molecules that selectively target AC1, but also caution that possible adverse reactions should be thoroughly studied.

The reviews and perspectives published in the Research Topic cover diverse topics related to AC structure and function. A systematic review by Shultz provides a deep analysis of how membrane-bound ACs evolved to their present structural arrangement. The article compares the different isoforms and provides detailed discussion on each AC domain. The perspective article by Ferreira et al. details the structure and functions of the soluble AC. The article also informs on how that isoform can be targeted for contraception, including possible dosing regimens and routes of administration. Three review articles in the Research Topic centered on the role of ACs on neuropsychiatric disorders. The review by Chen et al. focuses on the calcium-stimulated group 1 ACs. The authors outline their functions and discuss the available literature that links those AC isoforms with neuropsychiatric and neurodevelopmental diseases, such as depression and schizophrenia, as well as the therapeutic potential of targeting them. Tabakoff and Hoffman review the role played by AC7 in alcohol use disorder and depression. The manuscript discussions range from the molecular regulation of AC7 to genetic associations with neuropsychiatric disorders. The authors also discuss potential strategies for selectively modulating AC7. The third review article, by Schappi and Rasenick, centers on the relationship between Gα_s_ and depressive disorders. The authors provide comprehensive analyses of individual AC isoforms, cell signaling cascades, and the link between current antidepressant therapies and AC activity.

The articles in the topic present and discuss the latest findings and the different strategies being pursued and hypothesized for targeting AC isoforms to treat diseases. In addition to the different tools to study AC function that are identified and validated, the articles provide a framework for targeting individual AC isoforms and the contexts where AC modulation would be desired. Direct modulation of ACs remains an attractive path for the development of new therapies, however, there are challenges related to isoform selectivity and possible adverse reactions that need to be overcome. Overall, the articles in the present Research Topic provide a positive outlook for targeting AC isoforms but also caution about isoform selectivity and off-target effects. Additionally, the original research articles provide preclinical proof-of-concept for the use of AC isoform modulators and a framework for the development of novel small molecules that selectively target AC isoforms. Finally, the editors would like to thank all contributors and reviewers that made this Research Topic possible.
